# BA.5 bivalent booster vaccination enhances neutralization of XBB.1.5, XBB.1.16 and XBB.1.9 variants in patients with lung cancer

**DOI:** 10.1038/s41541-023-00779-8

**Published:** 2023-11-21

**Authors:** Rajesh M. Valanparambil, Lilin Lai, Margaret A. Johns, Meredith Davis-Gardner, Susanne L. Linderman, Tarrant Oliver McPherson, Andres Chang, Akil Akhtar, Estefany L. Bocangel Gamarra, Hayley Matia, Ashley A. McCook-Veal, Jeffrey Switchenko, Tahseen H. Nasti, Felicia Green, Manpreet Saini, Andreas Wieland, Benjamin A. Pinsky, Daniel Solis, Madhav V. Dhodapkar, Jennifer Carlisle, Suresh Ramalingam, Rafi Ahmed, Mehul S. Suthar

**Affiliations:** 1 Emory Vaccine Center, Emory University School of Medicine, Atlanta, GA USA; 2 Department of Microbiology and Immunology, Emory University, Atlanta, GA USA; 3 Department of Pediatrics, Emory University, Atlanta, GA USA; 4 Emory National Primate Center, Atlanta, GA USA; 5 Winship Cancer Institute of Emory University, Atlanta, GA USA; 6 Department of Biostatistics and Bioinformatics, Rollins School of Public Health, Emory University, Atlanta, USA; 7 Biostatistics Shared Resource, Winship Cancer Institute, Emory University, Atlanta, USA; 8 Department of Hematology and Medical Oncology, Emory University, Atlanta, GA USA; 9 Department of Otolaryngology, The Ohio State University, Columbus, OH USA; 10 Pelotonia Institute for Immuno-Oncology, The Ohio State University, Columbus, OH USA; 11 Department of Pathology, Stanford University School of Medicine, Stanford, CA USA

**Keywords:** Non-small-cell lung cancer, RNA vaccines

## Abstract

This study reports that most patients with NSCLC had a significant increase in the nAb response to the currently circulating Omicron variants after bivalent booster vaccination and had Ab titers comparable to healthy participants. Interestingly, though the durability of the nAb response persisted in most of the healthy participants, patients with NSCLC had significantly reduced nAb titers after 4–6 months of vaccination. Our data highlight the importance of COVID-19 bivalent booster vaccination as the standard of care for patients with NSCLC given the evolution of new variants of concern.

## Introduction

Monovalent vaccines containing the SARS-CoV-2 WA.1 (WT) spike mRNA have proven to be safe and effective in preventing serious illness in most patients with solid tumors^1,2^. However, Omicron variants have several mutations in the receptor binding domain (RBD) that enable them to evade WT vaccine-induced neutralizing antibodies (nAb). Administration of bivalent vaccines, which carry both WT and Omicron BA.5 spike mRNA, elicit nAb responses against Omicron variants in healthy individuals^3,4^. Previously, we and others have shown that some patients with non-small cell lung cancer (NSCLC) respond poorly to monovalent vaccines and fail to generate nAb titers against variants of concern^5,6^. Though bivalent booster vaccines are administered to cancer patients, the immune response in these patients is unknown. Here we examined the nAb responses in patients with NSCLC against WT and Omicron variants BA.5, BQ.1.1, XBB.1.5 and the recently evolved XBB.1.16 and XBB.1.9 variants.

## Results

Plasma was collected from 34 patients with NSCLC (41 samples) (Supplementary Table 1) and 12 healthy participants (Supplementary Table 2) and nAb titers against WT and Omicron variants BA.5, BQ1.1, XBB1.5, XBB1.16, and XBB1.9 were assessed (Supplementary Fig. 1 and Supplementary Table 3). Patients in the monovalent cohort (*n* = 11) had received 2 prior vaccine doses with an average time of 6–8 months since the last dose. Healthy participants and patients with NSCLC in the bivalent cohort had received 3 prior monovalent vaccine doses, with an average time of 10–12 months since the last vaccination (Supplementary Table 1).

**Fig. 1:**
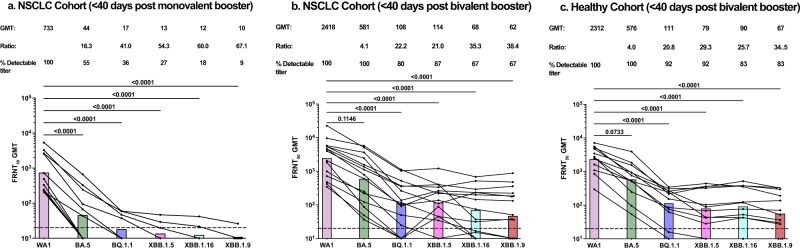
Neutralizing responses against the WA1/2020 strain and Omicron subvariants after monovalent and bivalent booster in patients with NSCLC and in healthy participants. Neutralization activity against the WA1/2020 strain of severe acute respiratory syndrome coronavirus 2 (SARS-CoV-2) and the Omicron subvariants BA.5, BQ1.1, XBB1.5, XBB1.16, and XBB1.9 in 11 patients with NSCLC after monovalent booster (**a**) 15 patients with NSCLC (**b**) and in 12 healthy participants (**c**) within 40 days of receiving the bivalent booster. Top of each panel shows the focus reduction neutralization test (FRNT50 [the reciprocal dilution of serum that neutralizes 50% of the input virus]) geometric mean titer (GMT) of neutralizing antibodies against the WA1/2020 strain and each Omicron subvariant, along with the ratio of the neutralization GMT against the WA1/2020 strain to that against each Omicron subvariant and the percentage of participants with detectable neutralizing antibody titers. The connecting lines between the variants represent matched serum samples. The horizontal dotted lines represent the limit of detection of the assay (FRNT50 GMT 20). The colored bars represent the FRNT50 GMT among the participants in the cohort. Figures show the percentage of detectable neutralizing titers, mean, and SEM.

After 40 days of the booster, all patients in the monovalent cohort had detectable nAb titers against the WT virus, however, the nAb response to all Omicron variants was significantly reduced (*P* < 0.0001). Only 18% and 9% of the patients in this cohort generated detectable nAb titers to the XBB1.16 and XBB1.9 variants respectively (Fig. 1a). Importantly, the nAb response increased significantly after the bivalent booster in patients with NSCLC (Supplementary Fig. 2). All patients in the bivalent cohort generated nAbs to the WT and BA.5 variant. More than 80% of the patients had nAb titers to the BQ.1.1 and XBB.1.5 variants while more than 65% of the patients had detectable nAb to the XBB.1.16 and XBB.1.9 variants. However, the nAb titers to BQ.1.1, XBB.1.5, XBB.1.16, and XBB.1.9 were significantly reduced compared to the WT virus (*P* < 0.0001) (Fig. 1b). Next, we evaluated the nAb response in the plasma of healthy participants after bivalent booster. All healthy participants had detectable nAb to the WT and BA.5 viruses, while 92% of the cohort made nAb to BQ.1.1 and XBB.1.5, 83% had nAb titers to XBB.1.16 and XBB.1.9 variants. Similar to the patients with NSCLC in the bivalent cohort, the neutralizing activity against currently circulating XBB variants was significantly reduced compared to the WT virus in the healthy cohort (*P* < 0.0001) (Fig. 1c). Interestingly, compared to the healthy participants there was no significant difference in the nAb titers to any virus in patients with NSCLC after the bivalent booster (Supplementary Fig. 3).

**Fig. 2:**
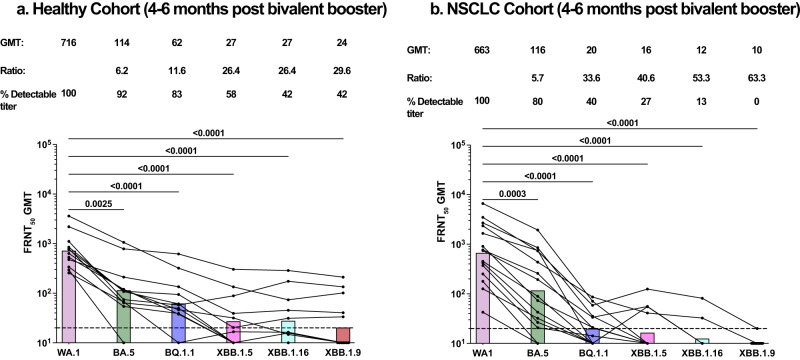
Durability of nAb titers in healthy participants and in patients with NSCLC 4–6 months after bivalent booster. nAb titers to the WA1/2020 strain and the Omicron subvariants BA.5, BQ1.1, XBB1.5, XBB1.16, and XBB1.9 in 12 healthy participants (**a**) and 15 patients with NSCLC (**b**) at 4–6 months after receiving the bivalent booster. Top of each panel shows the focus reduction neutralization test (FRNT50 [the reciprocal dilution of serum that neutralizes 50% of the input virus]) geometric mean titer (GMT) of neutralizing antibodies against the WA1/2020 strain and each Omicron subvariant, along with the ratio of the neutralization GMT against the WA1/2020 strain to that against each Omicron subvariant and the percentage of participants with detectable neutralizing antibody titers. The connecting lines between the variants represent matched serum samples. The horizontal dotted lines represent the limit of detection of the assay (FRNT50 GMT 20). The colored bars represent the FRNT50 GMT among the participants in the cohort. Figures show the percentage of detectable neutralizing titers, mean, and SEM.

Next, we evaluated the durability of nAb response after bivalent booster in healthy participants and patients with NSCLC. Totally, 4–6 months after the bivalent booster vaccination, we observed a reduction in the nAb titers to the WT and the Omicron variants in the healthy participants. While all healthy participants had nAbs against the WT virus, more than 90% and 80% of participants had nAbs against BA.5 and BQ1.1 respectively. More than 55% of the participant had nAbs against the XBB1.5 variant and 42% of the cohort had detectable nAbs against XBB1.16 and XBB1.9 variants (Fig. 2a). Interestingly, the nAb titers in the healthy cohort was significantly reduced after 4–6 months after booster compared to the titers observed within 40 days of booster (WT *P* < 0.0029, BA.5 *P* < 0.0145, BQ.1.1 *P* < 0.1428, XBB.1.5 *P* < 0.0490, XBB.1.16 *P* < 0.0416 and XBB.1.9 *P* < 0.0530) (Supplementary Fig. 4).

Interestingly the decrease in the durability of nAb response after the bivalent booster was more significant in the NSCLC cohort compared to the healthy cohort. Though there was no difference in the nAb titers in the NSCLC cohort compared to the healthy cohort within 40 days after the bivalent booster, the nAb titers to the BQ1.1 (*P* < 0.0123), XBB1.16 (*P* < 0.0095) and XBB1.9 (*P* < 0.0267) were significantly reduced in the NSCLC bivalent booster cohort compared to the healthy cohort 4–6 months post booster vaccination (Supplementary Fig. 5). In the NSCLC patient cohort, we observed a significant reduction in the nAb titers to the Omicron variants compared to the WT strain (*P* < 0.0001) 4–6 months after the bivalent booster. Though all patients had detectable nAb against the WT strain, only 27% and 13% of the cohort had detectable nAb titers to XBB.1.5 and XBB.1.16 variants, respectively. None of the patients in the NSCLC cohort had detectable nAb titers to the XBB1.9 variants (Fig. 2b). The durability of nAb response was also significantly lower in the NSCLC patients at 4–6 months compared to 40 days of the bivalent booster (WT *P* < 0.0164, BA.5 *P* < 0.0161, BQ.1.1 *P* < 0.0024, XBB.1.5 *P* < 0.0002, XBB.1.16 *P* < 0.0002 and XBB.1.9 *P* < 0.0002) (Supplementary Fig. 6).

Next, we examined if cancer treatment modality and patient demographics of our monovalent and bivalent cohorts influenced our findings discussed above. Age, race, gender, cancer stage, and vaccine type were not statistically different between the cohorts (Supplementary Table 1). As some patients were actively receiving cancer therapy, we also determined whether different cancer therapies influenced the antibody response to vaccination. Patients in our cohorts were divided into different subsets based on their cancer therapy (chemotherapy, immunotherapy, targeted therapy, combination therapy (immunotherapy and chemotherapy), and patients under surveillance. There was no significant bias toward a specific therapy in either of our cohorts which corroborates previous studies^5,6^ that the type of cancer therapy that the patients received does not influence the nAb response to vaccination (Supplementary Table 1).

## Discussion

Here, we studied the antibody response against SARS-CoV-2 WT and Omicron variants following bivalent booster vaccination in NSCLC patients compared to healthy participants. Importantly, only 9–27% of patients had detectable nAb titers to the XBB sub-variants after monovalent booster vaccination. Given that XBB sub-variants are currently the predominant circulating variants in the US, this poses a higher risk of infection in patients who have only received the monovalent booster. nAb titers against Omicron variants, including the XBB1.5, XBB1.16, and XBB1.9 were significantly higher early after bivalent booster vaccination. Interestingly, NSCLC patients had similar levels of nAb titers compared to the healthy participants. About 87% of patients with NSCLC in our bivalent cohort had detectable XBB.1.5 nAb titers while more than 65% of the patients had detectable nAb titers to XBB.1.16 and XBB.1.9 variants. Importantly, the durability of nAb titers to the Omicron variants was significantly reduced in patients with NSCLC 4–6 months after bivalent booster. This should be taken into consideration when designing vaccine regimens for cancer patients. Interestingly, the type of cancer therapy did not influence the nAb response after either monovalent or bivalent vaccination. More detailed studies with larger cohorts are necessary to validate this observation. In conclusion, though XBB sub-variant nAb titers were significantly higher after bivalent vaccination, these titers were significantly reduced after 4–6 months. This highlights the need to improve the currently available vaccine strategies. Since bivalent vaccines produce significant live virus nAb responses against the currently circulating variants, bivalent booster vaccination should be recommended for patients with NSCLC.

Our study does have some limitations. First, the cohort size of our study is relatively small and all the patients in the study are from a single institution. Second, the median age of patients in the NSCLC cohort is higher than the healthy participant cohort. As Ab titers in older individuals, are significantly lower than the younger individuals^5,7^, the higher median age of our NSCLC cohorts could have contributed to the rapid waning of nAb response in patients with NSCLC. Third, the bivalent booster vaccinees have received one more dose of vaccination compared to the monovalent booster recipients.his could have influenced the data presented here. Another limitation of this study is that, our study also lacks T-cell analysis.

## Methods

### Cohort of patients with NSCLC

Peripheral blood samples from patients with NSCLC were collected at Winship Cancer Institute following written informed consent approved by the Institutional Review Board at Emory University. Blood samples were processed to isolate plasma and mononuclear cells. The patient demographics for all 34 patients with NSCLC enrolled in the study are in Supplementary Table 1. Peripheral blood samples were collected for analyzing antibody responses from all enrolled NSCLC patients. Of the enrolled patients, samples were collected from 11 monovalent booster recipients and 15 bivalent booster recipients within 40 days of respective booster vaccination. Samples from 15 patients with NSCLC were collected within 4–6 months of bivalent booster.

### Cells and viruses

Vero-TMPRSS2 cells were cultured in a complete DMEM medium consisting of 1× DMEM (VWR, #45000-304), 10% FBS, 2 mM l-glutamine, and 1× antibiotic as previously described^8^. nCoV/USA_WA1/2020 (WA/1), closely resembling the original Wuhan strain, was propagated from an infectious SARS-CoV-2 clone as previously described^9^. icSARS-CoV-2 was passed once to generate a working stock. Omicron subvariants were isolated from residual nasal swabs: BA.5 isolate (EPI_ISL_13512579), provided by Dr. Richard Webby (St Jude Children’s Research Hospital), BQ.1.1 isolate (EPI_ISL_15196219), XBB.1.5 isolate (EPI_ISL_16026423) was provided by Dr. Andrew Pekosz and passaged from a stock. XBB.1.9 isolate (EPI_ISL_17417339), and XBB.1.16 isolate (EPI_ISL_17417328) were provided by Dr. Benjamin Pinsky (Stanford University). All variants were plaque purified and propagated once in VeroE6-TMPRSS2 cells to generate working stocks.

### Focus reduction neutralization assay

FRNT assays were performed as previously described^10^. Briefly, samples were diluted at 3-fold in 8 serial dilutions using DMEM in duplicates with an initial dilution of 1:10 in a total volume of 60 μl. Serially diluted samples were incubated with an equal volume of SARS-CoV-2 (100–200 foci per well) at 37 °C for 1 h in a round-bottomed 96-well culture plate. The antibody-virus mixture was then added to Vero cells and incubated at 37 °C for 1 h. Post-incubation, the antibody-virus mixture was removed and 100 µl of prewarmed 0.85% methylcellulose overlay was added to each well. Plates were incubated at 37 °C for 18–40 h, and the methylcellulose overlay was removed and washed six times with PBS. Cells were fixed with 2% paraformaldehyde in PBS for 30 min. Following fixation, plates were washed twice with PBS, and permeabilization buffer (0.1% BSA, 0.1% Saponin in PBS) was added to permeabilize cells for at least 20 min. Cells were incubated with an anti-SARS-CoV spike primary antibody directly conjugated to Alexa Fluor-647 (CR3022-AF647) overnight at 4 °C. Cells were then washed twice with 1× PBS and imaged on an ELISPOT reader (CTL Analyzer).

### Quantification and statistical analysis

Antibody neutralization was quantified by counting the number of foci for each sample using the Viridot program^11^ The neutralization titers were calculated as follows: 1 − (ratio of the mean number of foci in the presence of sera and foci at the highest dilution of the respective sera sample). Each specimen was tested in duplicate. The FRNT-50 titers were interpolated using a 4-parameter nonlinear regression in GraphPad Prism 9.2.0. Samples that do not neutralize at the limit of detection at 50% are plotted at 20 and used for geometric mean and fold-change calculations. Statistical analysis was performed using GraphPad Prism V9. Statistical differences were assessed using either two-tailed paired Student’s *t*-test, unpaired *t*-test, or one-way ANOVA with multiple comparisons. *P* values of <0.05 were considered significant.

### Reporting summary

Further information on research design is available in the Nature Research Reporting Summary linked to this article.

### Supplementary information

Supplementary Material

REPORTING SUMMARY

## Data Availability

Raw data is available on request to the corresponding author.
