# Supplementary material for: BA.5 bivalent booster vaccination enhances neutralization of XBB.1.5, XBB.1.16 and XBB.1.9 variants in patients with lung cancer
Source: NPJ Vaccines. 2023 Nov 21;8:179. doi: 10.1038/s41541-023-00779-8 (PMC10663480; doi:10.1038/s41541-023-00779-8)
Supplement: Supplementary file 1 — Supplementary Material [file 41541_2023_779_MOESM1_ESM.pdf]

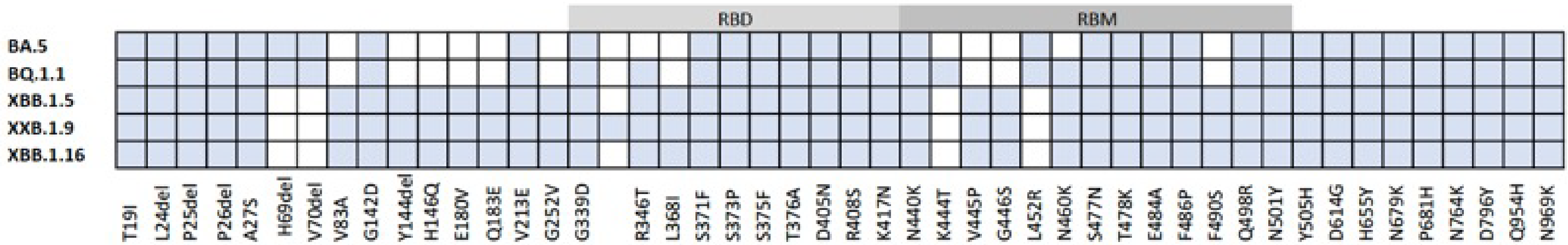

Supplementary Figure 1. Alignment of Spike protein sequence for the different variants analyzed in this study. White boxes indicate wild-type sequence while the colored box indicates amino acid substitution labeled below. Receptor binding domain (RBD) and receptor binding motif (RBM) are indicated above.

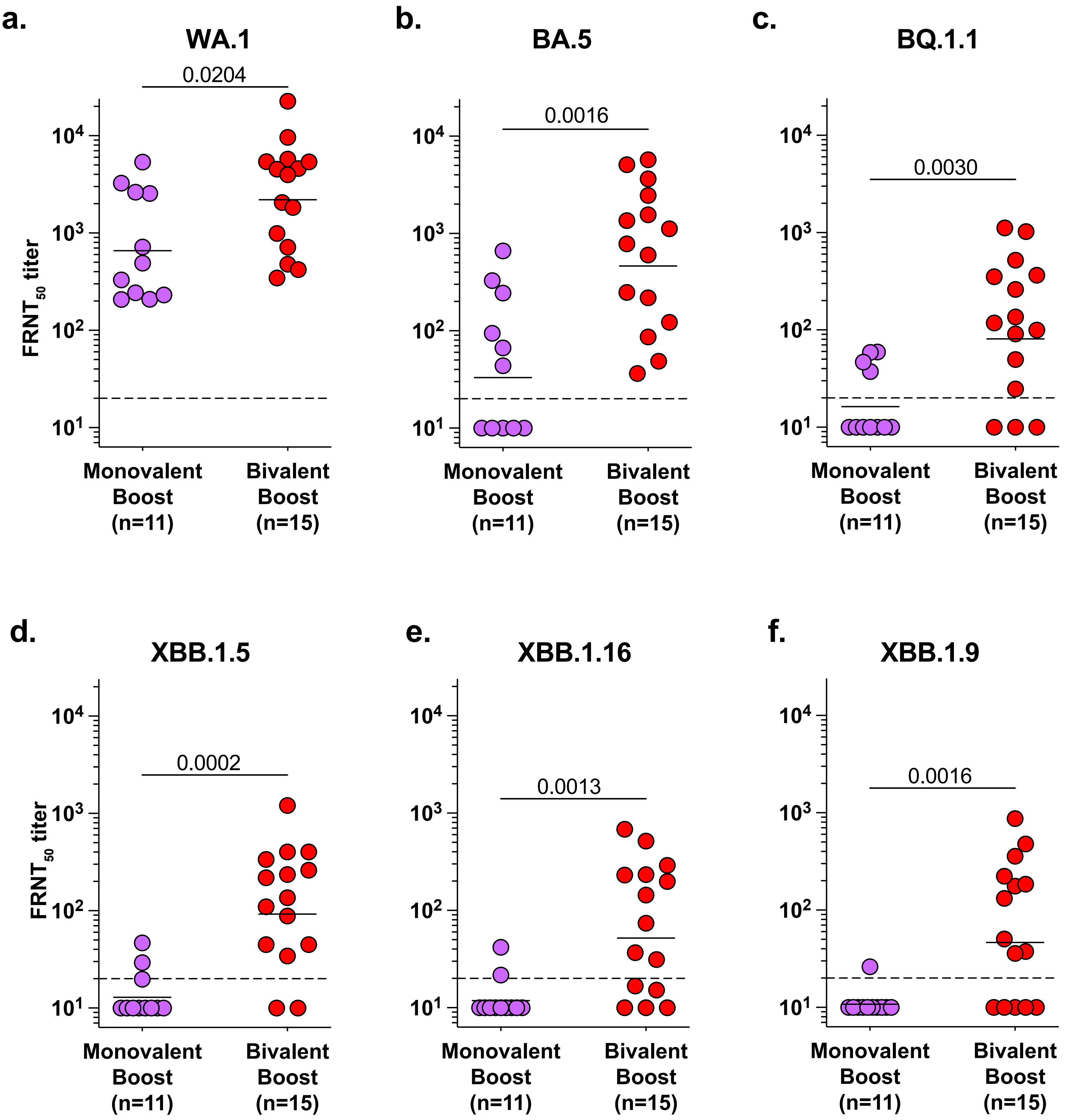

Supplementary figure 2. nAb response against the WT strain and Omicron Subvariants within 40 days of monovalent and bivalent booster in patients with NSCLC.
nAb titers against WT strain (A), BA.5 (B), BQ1.1 (C), XBB1.5 (D), XBB1.16 (E) and XBB1.9 (F) in patients with NSCLC within 40 days of monovalent booster compared to the bivalent booster vaccination. Figures shows the mean and SEM.

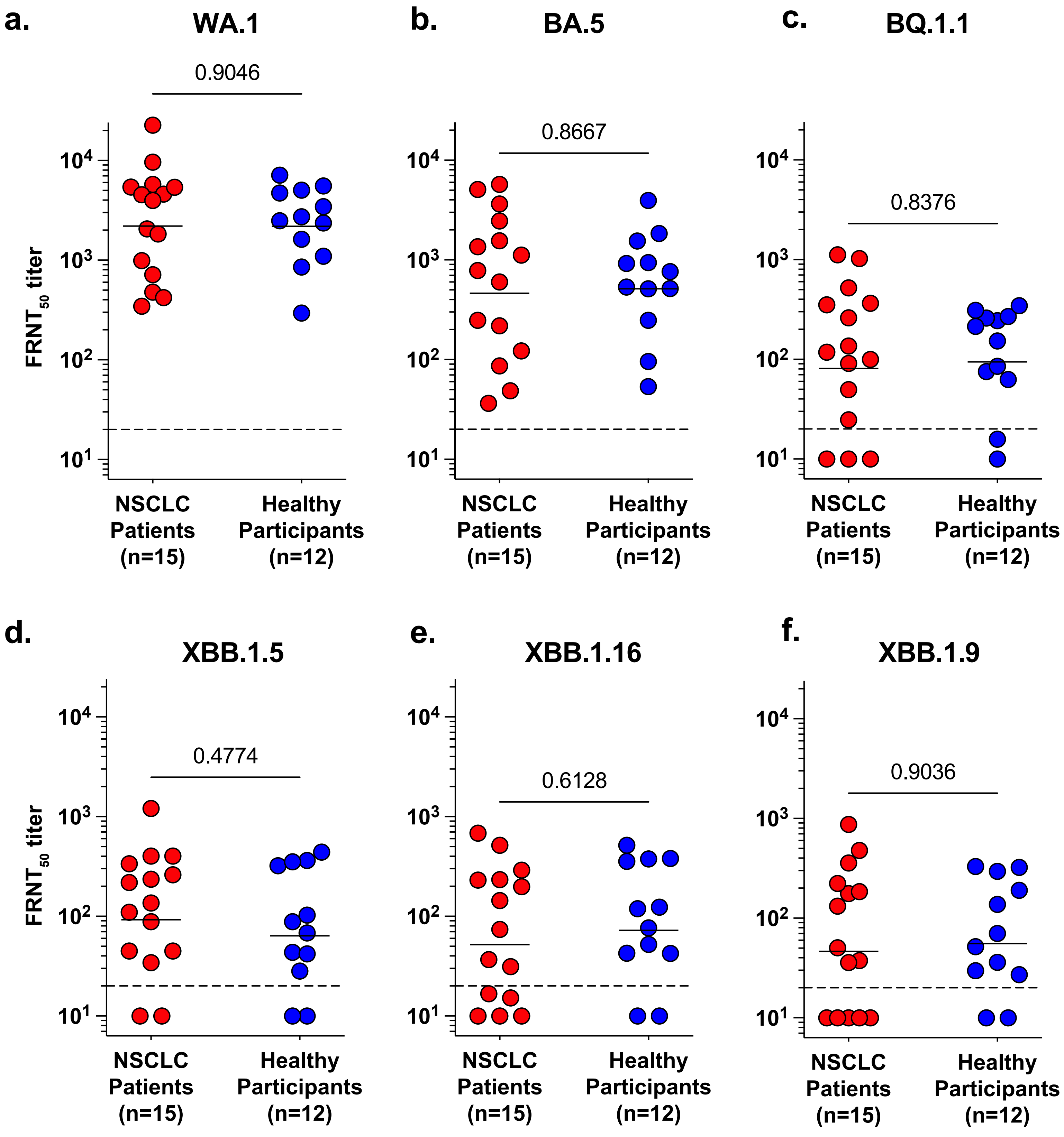

Supplementary Figure 3. nAb response against the WT strain and Omicron Subvariants within 40 days of bivalent booster in patients with NSCLC and in healthy participants.
nAb titers against WT strain (A), BA.5 (B), BQ1.1 (C), XBB1.5 (D), XBB1.16 (E) and XBB1.9 (F) in patients with NSCLC compared to healthy participants within 40 days of bivalent booster vaccination.
Figures shows the mean and SEM.

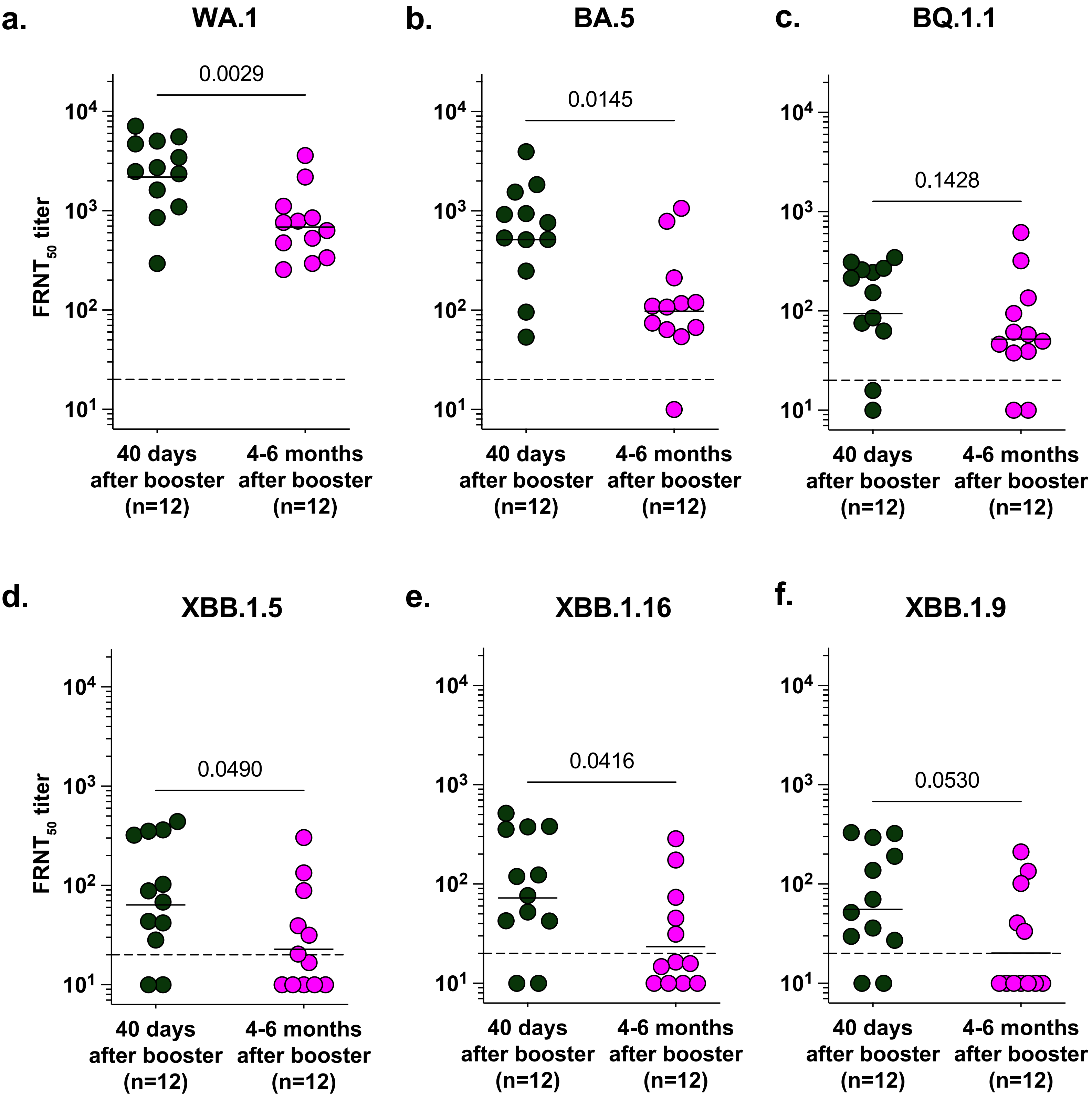

Supplementary Figure 4. nAb response in healthy recipients against the WT strain and Omicron Subvariants within 40 days compared to 4-6 months after bivalent booster

nAb titers against WT strain (A), BA.5 (B), BQ1.1 (C), XBB1.5 (D), XBB1.16 (E) and XBB1.9 (F) in healthy participants within 40 days of bivalent booster vaccination compared to 4-6 months after booster.

Figures shows the mean and SEM.

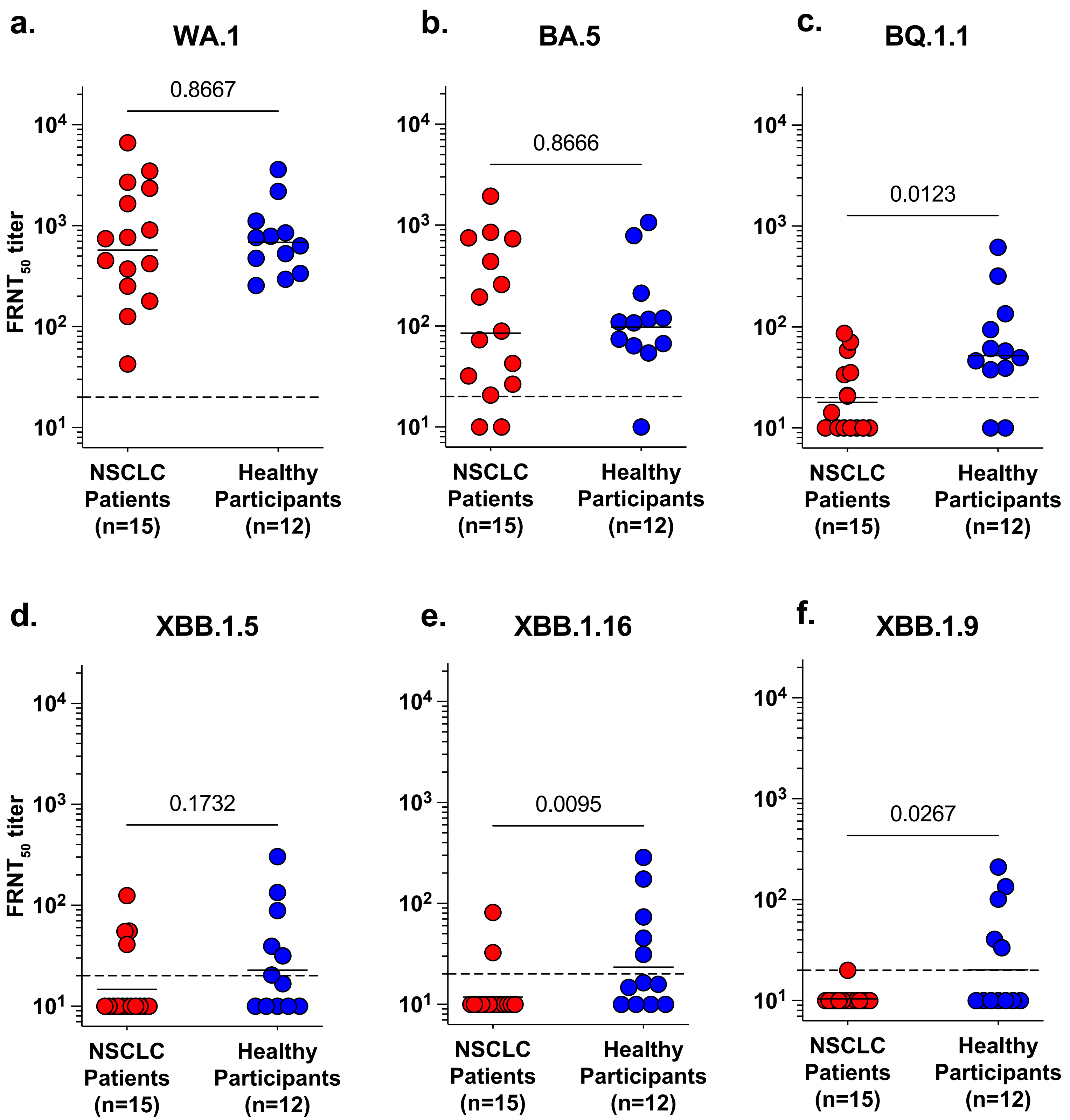

Supplementary Figure 5. nAb response against the WT strain and Omicron Subvariants after 4-6 months of bivalent booster in patients with NSCLC and in healthy participants.
nAb titers against WT strain (A), BA.5 (B), BQ1.1 (C), XBB1.5 (D), XBB1.16 (E) and XBB1.9 (F) in patients with NSCLC compared to healthy participants 4-6 months after bivalent booster vaccination.
Figures shows the mean and SEM.

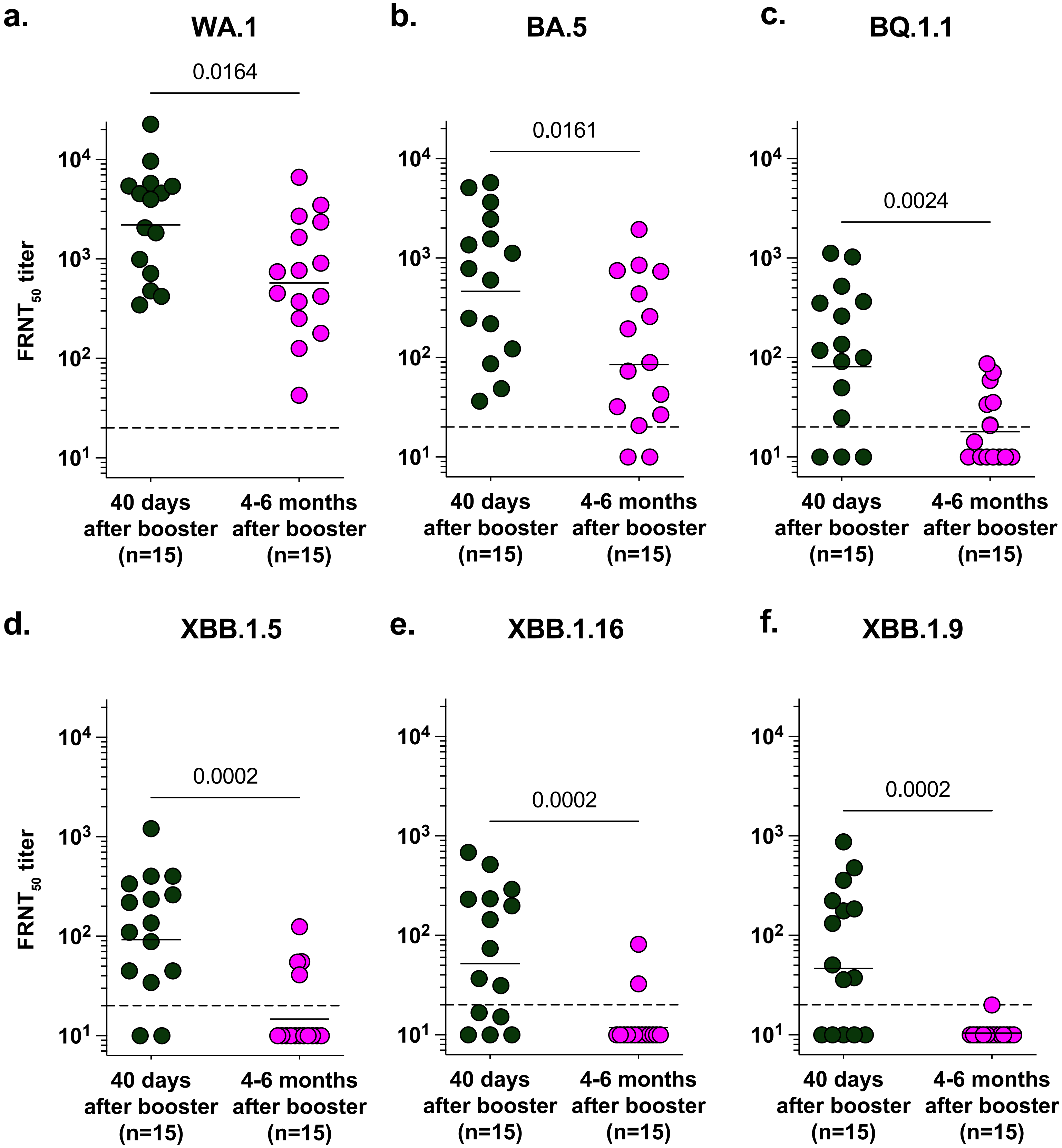

Supplementary Figure 6. nAb response in patients with NSCLC against the WT strain and Omicron Subvariants within 40 days compared to 4-6 months after bivalent booster

nAb titers against WT strain (A), BA.5 (B), BQ1.1 (C), XBB1.5 (D), XBB1.16 (E) and XBB1.9 (F) in patients with NSCLC within 40 days of bivalent booster vaccination compared to 4-6 months after booster.

Figures shows the mean and SEM.

Supplementary Table 1. Characteristics of Samples from Patients with NSCLC

Variable	Overall<br>N = 32[1]	Monovalent<br>N = 9[1]	Bivalent[2]<br>N = 21[1]	Both<br>N = 2[1]	p-value[3,4]
**Sex**					0.43
Female	15 (47%)	6 (67%)	9 (43%)	0 (0%)	
Male	17 (53%)	3 (33%)	12 (57%)	2 (100%)	
**Age at first vaccine**	68 (63, 74)	65 (63, 79)	70 (63, 72)	66 (65, 67)	0.74
**Race**					>0.99
Asian	1 (3.1%)	0 (0%)	1 (4.8%)	0 (0%)	
Black or African American	9 (28%)	3 (33%)	6 (29%)	0 (0%)	
White	21 (66%)	6 (67%)	13 (62%)	2 (100%)	
**Ethnicity**					>0.99
Hispanic	1 (3.1%)	0 (0%)	1 (4.8%)	0 (0%)	
Non-Hispanic	29 (91%)	9 (100%)	18 (86%)	2 (100%)	
**Smoking Status**					>0.99
N	11 (34%)	3 (33%)	7 (33%)	1 (50%)	
Y	21 (66%)	6 (67%)	14 (67%)	1 (50%)	
**Stage**					0.16
I/Ia/Ib/Ic	8 (25%)	3 (33%)	5 (24%)	0 (0%)	
II/IIa/IIb/IIc	7 (22%)	4 (44%)	2 (9.5%)	1 (50%)	
III/IIIa/IIIb/IIIc	7 (22%)	1 (11%)	6 (29%)	0 (0%)	
IV/IVa/IVb/IVc	9 (28%)	1 (11%)	7 (33%)	1 (50%)	
**Therapy**					
Chemotherapy	2 (6.2%)	2 (22%)	0 (0%)	0 (0%)	0.08
Immunotherapy	9 (28%)	3 (33%)	5 (24%)	1 (50%)	0.67
Targeted	7 (22%)	1 (11%)	6 (29%)	0 (0%)	0.39
Surveillance	10 (31%)	2 (22%)	7 (33%)	1 (50%)	0.68
Combination	6 (19%)	1 (11%)	5 (24%)	0 (0%)	0.64

[1]n (%); Median (IQR)

[2]There were 7 patients with samples for both <40 days and 4-6 months post-vaccination

[3]Fisher's exact test; Kruskal-Wallis rank sum test

[4]Only reflects comparison between Monovalent and Bivalent due to small samples size in Both group

Supplemental Table 2: Demographic information for healthy cohort

Variable	N = 12[1]
**Sex**	
F	7 (58%)
M	5 (42%)
**Age at vaccination**	39 (34, 46)
**Exposure Status**	
Recovered	2 (17%)
Naïve	10 (83%)
**Vaccine Used**	
Moderna	3 (25%)
Pfizer	1 (8.3%)
Unknown	8 (67%)
**Time from Vaccination**	20 (17, 23)

[1]n (%); Median (IQR)

Supplemental Table 3: Amino acid substitutions in spike protein of variants used in the study.

Variant	Virus Name	GISAID	Amino Acid Substitutions
**BA.5**	hcov-19/USA/MD/HP30386/2022	EPI_ISL_13512579	T19I, L24del, P25del, P26del, A27S, H69del, V70del, T76I, G142D, V213G, G339D, S371F, S373P, S375F, T376A, D405N, R408S, K417N, N440K, L452R, S477N, T478K, E484A, F486V, Q498R, N501Y, Y505H, D614G, H655Y, N679K, P681H, N764K, D796Y, Q954H, N969K
**BQ.1.1**	hCoV-19/USA/CA-Stanford-106_S04/2022	EPI_ISL_15196219	T19I, L24del, P25del, P26del, A27S, H69del, V70del, G142D, V213G, G339D, R346T, S371F, S373P, S375F, T376A, D405N, R408S, K417N, N440K, K444T, L452R, N460K, S477N, T478K, E484A, F486V, Q498R, N501Y, Y505H, D614G, H655Y, P681H, N679K, N764K, D796Y, Q954H, N969K
**XBB.1.5**	hCoV-19/USA/MD-HP40900-PIDYSWHNUB/2022	EPI_ISL_16026423	T19I, L24del, P25del, P26del, A27S, V83A, G142D, Y144del, H146Q, Q183E, V213E, G252V, G339H, R346T, L368I, S371F, S373P, S375F, T376A, D405N, R408S, K417N, N440K, V445P, G446S, N460K, S477N, T478K, E484A, F486P, F490S, Q498R, N501Y, Y505H, D614G, H655Y, N679K, P681H, N764K, D796Y, Q954H, N969K
**XBB.1.16**	hCoV-19/USA/CA-Stanford-139_S23/2023	EPI_ISL_17417328	T19I, L24del, P25del, P26del,A27S, V83A, G142D,Y144del ,H146Q, E180V, Q183E, V213E, G252V, G339H, R346T, L368I, S371F, S373P, S375F, T376A, D405N, R408S, K417N, N440K, V445P, G446S, N460K, S477N, T478R, E484A, F486P, F490S, Q498R,

			N501Y, Y505H, D614G, H655Y, N679K, P681H, N764K, D796Y, Q954H, N969K
**XBB.1.9**	hCoV-19/USA/CA-Stanford-106_S04/2022	EPI_ISL_1741733 9	T19I, L24del, P25del, P26del, A27S, V83A, G142D, Y144del, H146Q,Q183E, V213E, G252V, G339H, A344S, R346T, L368I, S371F, S373P, S375F, T376A, D405N, R408S, K417N, N440K, V445P, G446S, N460K, S477N, T478K, E484A, F486P, F490S, Q498R, N501Y, Y505H, D614G, H655Y, N679K, P681H, N764K, D796Y, Q954H, N969K
